# Proprioceptive Sonomyographic Control: A novel method for intuitive and proportional control of multiple degrees-of-freedom for individuals with upper extremity limb loss

**DOI:** 10.1038/s41598-019-45459-7

**Published:** 2019-07-01

**Authors:** Ananya S. Dhawan, Biswarup Mukherjee, Shriniwas Patwardhan, Nima Akhlaghi, Guoqing Diao, Gyorgy Levay, Rahsaan Holley, Wilsaan M. Joiner, Michelle Harris-Love, Siddhartha Sikdar

**Affiliations:** 1 Department of Computer Science, George Mason University, Fairfax, VA 22030 USA; 2 Department of Bioengineering, George Mason University, Fairfax, VA 22030 USA; 3 Center for Adaptive Systems of Brain-Body Interactions, George Mason University, Fairfax, VA 22030 USA; 4 Department of Statistics, George Mason University, Fairfax, VA 22030 USA; 5 Infinite Biomedical Technologies, Baltimore, MD 21202 USA; 6 MedStar National Rehabilitation Hospital, Washington, DC 20010 USA

**Keywords:** Motor control, Biomedical engineering

## Abstract

Technological advances in multi-articulated prosthetic hands have outpaced the development of methods to intuitively control these devices. In fact, prosthetic users often cite "difficulty of use" as a key contributing factor for abandoning their prostheses. To overcome the limitations of the currently pervasive myoelectric control strategies, namely unintuitive proportional control of multiple degrees-of-freedom, we propose a novel approach: *proprioceptive sonomyographic* *control*. Unlike myoelectric control strategies which measure electrical activation of muscles and use the extracted signals to determine the velocity of an end-effector; our sonomyography-based strategy measures mechanical muscle deformation directly with ultrasound and uses the extracted signals to proportionally control the position of an end-effector. Therefore, our sonomyography-based control is congruent with a prosthetic user’s innate proprioception of muscle deformation in the residual limb. In this work, we evaluated *proprioceptive sonomyographic control* with 5 prosthetic users and 5 able-bodied participants in a virtual target achievement and holding task for 5 different hand motions. We observed that with limited training, the performance of prosthetic users was comparable to that of able-bodied participants and thus conclude that *proprioceptive sonomyographic control* is a robust and intuitive prosthetic control strategy.

## Introduction

Currently, there are approximately 50,000 individuals living with upper limb loss in the US^1^. Upper extremity amputations most commonly occur in working age adults as a result of trauma^1,2^ and frequently affects the dominant extremity^3^. This significantly impacts activities of daily living, and contributes to overuse injuries to the sound limb^4^. The most common upper extremity amputation occurs at the transradial level (57%)^3^. A large proportion of individuals with upper limb loss discontinue the use of their prosthesis^5–7^. Prosthetic non-wear has been reported in 20% of individuals with upper limb loss and rejection rates for upper limb prosthetic users range from 35%–45% for myoelectric and cable controlled systems^5^. Currently, commercially-available advanced prosthetic hands, while capable of dexterous grips, lack intuitive control. Dissatisfaction with prosthesis technology is highly associated with rejection^5^. Most non-users (88%) reported the systems as being “too difficult or tiring” to use^8^. However, 74% of those who have abandoned their upper limb prostheses stated that they would reconsider prosthetic use if technological advancements were made to improve their functionality and usability^8^. Therefore, there is a significant unmet need for better technological solutions to benefit individuals with upper limb loss.

Despite enormous investment of resources in the development of new multi-articulated upper limb prostheses, one of the limiting factors remains how to accurately and intuitively control these advanced hand and wrist systems. For the past 50 years, electromyography has been the predominant method for sensing muscle activation, primarily utilizing surface electromyography (sEMG) electrodes. Decades of research have gone into improving myoelectric control technology for upper extremity prostheses. Yet, a recent study found that inertial measurement units on the foot are more effective in controlling the state-of-the-art multi-articulated DEKA arm compared to the most sophisticated myoelectric control method^9^.

A fundamental challenge with sEMG signals is the poor amplitude resolution and low signal-to-noise, especially with dry electrodes, typically used in prosthetic applications^10–12^, making it challenging for users to accurately achieve multiple graded levels of sEMG amplitude. Thus, in the most commonly used myoelectric control strategy, called direct control (DC), sEMG amplitude is thresholded to drive joint velocity instead of joint position. Velocity control limits the ability to achieve dexterous manipulation of the terminal device. Thus, DC users grip objects with excessive force and velocity as compared to able-bodied individuals^13^. The artificial velocity control signal is not congruent with proprioceptive feedback from residual muscles and is nonintuitive. Therefore, many users prefer body powered hooks over myoelectric systems as the cable tension provides congruent sensory feedback for terminal device position and thus is more intuitive^8,14^. Even with more sophisticated grasp decoding algorithms using pattern recognition with multiple electrodes^15–21^, the ability to obtain graded proportional control is limited by the fundamental challenges with sEMG amplitude resolution. Other invasive strategies, such as implantable myoelectric systems^22–24^, targeted muscle reinnervation^25^ and peripheral implant^26,27^ strategies, can produce more robust graded signals. Implanted devices and surgical procedures are associated with risk and side effects. While there is interest in these invasive technologies, users still prefer noninvasive methods^28^. Thus, there continues to be a need for a robust noninvasive strategy that can provide intuitive real-time proprioceptive control over multiple degrees-of-freedom to enable prosthetic users to make full use of advanced commercial hands.

In recent years, sonomyography, or ultrasound-based sensing of mechanical muscle contractions, has been actively pursued as an alternative to myoelectric control^29^. Ultrasound imaging provides a non-invasive sensing modality that can spatially resolve individual muscles, including those deep inside the tissue, and detect dynamic activity within different functional compartments in real-time. In fact, sonomyography has been shown to be useful for detecting individual finger positions^30,31^, along with other complex muscle deformation patterns^32^. Previous research has also shown that ultrasound techniques could be used for real-time classification of hand movements by predicting forearm muscle deformation patterns in able-bodied individuals^29,31^ as well as individuals with transradial amputation^33^.

In this paper, we propose a new sonomyographic control strategy to classify volitional motion intent and further extend our paradigm to enable intuitive, position-based proportional control over multiple degrees-of-freedom. Since our strategy relies on mechanical muscle contractions and relaxations that are congruent with underlying proprioceptive feedback in the residual limb, we refer to our strategy as *proprioceptive sonomyographic control*. We validated our techniques on able-bodied participants and applied it to five individuals with upper-extremity limb loss, one of whom had a congenital limb deficiency. We asked the participants to perform predefined hand motions while ultrasound images of their forearm muscle movements were captured using a portable, commercial ultrasound system. We extracted representative ultrasound images from the collected ultrasound data to build a training dictionary, and performed leave-one-out validation to quantify prediction accuracy using a supervised learning algorithm using k-nearest neighbors. Participants were then asked to perform the same hand motions in real-time while being shown an on-screen cursor that moved up or down in proportion to their muscle contract and relaxation respectively. A series of targets were presented at different levels of motion completion and the participant’s ability to perform graded control to reach these targets using forearm muscle deformation was measured. The goals of this work were, 1) to determine the ability of individuals with upper-extremity limb loss to perform different hand motions using our *proprioceptive sonomyographic control* strategy, with minimal training and 2) to determine the accuracy and stability with which individuals with limb loss can perform these motions in a graded manner to different levels of motion completion.

## Methods

### Subjects and experimental setup

We recruited four individuals with unilateral and one bilateral, upper-extremity amputation at the MedStar National Rehabilitation Hospital (Washington DC, USA) and George Mason University (Fairfax VA, USA). All of the individuals with limb loss were at the time using electrically powered, myoelectric prostheses with varying levels of proficiency. Subject-specific amputation details and demographics are available in Table 1. Five able-bodied participants were also recruited and served as a control group for this study. Demographics for able-bodied participants are listed in Table 2. All experiments described in this work were approved by the George Mason University Institutional Review Board and MedStar National Rehabilitation Hospital Institutional Review Board and performed in accordance with relevant guidelines and regulations. All participants provided written, informed consent prior to participating in the study, and were also compensated for their participation.

**Table 1:** Demographics and amputation details of prosthetic users.

Subject ID	Sex	Age	Years since amputation	Amputation type	Amputation level and side	Phantom limb	Phantom pain
*Am1*	M	68	50	Traumatic	Transradial (L*)	Yes	No
*Am2*	M	—	—	Congenital	Transradial (L*)	No	No
*Am3*	M	56	46	Traumatic	Transradial (R*)	No	No
*Am4*	M	30	7.5	Traumatic	Wrist disarticulation (L*), Transradial (R)	Yes (L*) Yes (R)	Yes (L*), No (R)
*Am5*	M	38	1.5	Traumatic	Transradial (R*)	No	No

(L) and (R) indicate amputation of left or right arm respectively and *indicates arm used for this study.

**Table 2:** Demographics of able-bodied participants.

Subject ID	Age	Sex	Dominant arm
*Ab1*	29	M	Right
*Ab2*	26	M	Right
*Ab3*	28	M	Right
*Ab4*	25	F	Left
*Ab5*	24	M	Right

**Figure 1:**
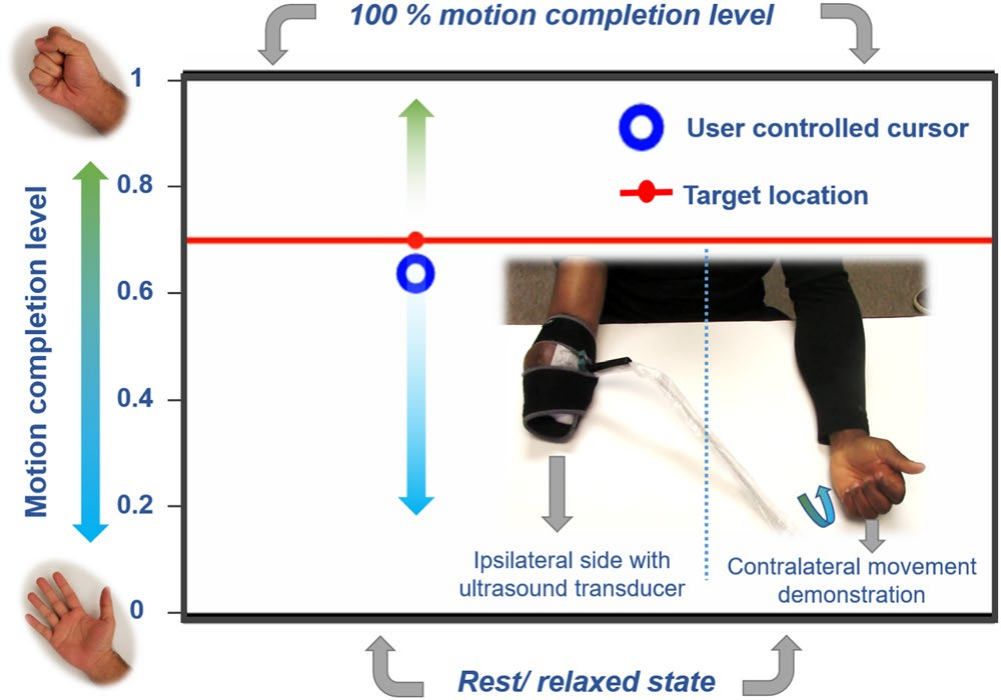
Photograph of the experimental setup showing an individual with transradial amputation instrumented with an ultrasound transducer on the residuum (inset). The interface for the target holding motion control task described in experiment 2 shows the target position, movement bounds and the cursor position, which is controlled by muscle deformations in the subject’s residuum. Participants with unilateral amputation were asked to demonstrate the perceived motion using their contralateral intact limb.

For the entire course of this study, all participants were seated upright with their forearm comfortably supported and their elbow below the shoulder to minimize fatigue. Subjects were instrumented with a clinical ultrasound system (Terason uSmart 3200T, Terason) connected to a low-profile, high-frequency, linear, 16HL7 transducer as shown in Fig. 1. The imaging depth was set to 4 cm without focusing. For individuals with limb loss, the transducer was positioned on the residuum below the elbow (see Fig. 1), such that all individual phantom finger movements resulted in considerable movement in the field-of-view. For able-bodied participants, the transducer was manually positioned on the volar aspect of the dominant forearm, approximately 4 cm–5 cm from the olecranon process in order to image both the deep and superficial flexor muscle compartments. Additionally, able-bodied participants were asked to place their forearm inside an opaque enclosure that prevented direct observation of arm and hand movements below the elbow. This ensured that able-bodied participants relied on their kinesthetic sense to perform all motion control tasks. The transducer was then secured in a custom-designed probe holder and held in place with a stretchable cuff as shown in Fig. 1 (inset). Ultrasound image sequences from the clinical ultrasound system were acquired and transferred to a PC (Intel Core i7-7700HQ, 16GB RAM, 4GB NVIDIA GeForce GTX 1040Ti) in real-time using a USB based video grabber (DVI2USB 3.0, Epiphan Systems, Inc.). The captured screen was then downscaled and cropped to (100 × 140 pixels) to include only the relevant ultrasound image. We typically obtained frame rates higher than 15 fps with this setup. The acquired image frame was then processed in MATLAB (The MathWorks, Inc.) using custom-developed algorithms as described in the following section.

### Control algorithms

Following probe placement, the participants underwent an initial training phase during which they performed repeated iterations of a set of motions (*power grasp, wrist pronation, thumb flexion, index flexion, key grasp, tripod, point*), one motion at a time. Movements were timed to a metronome such that the participant first transitioned from *rest* to the end state of the selected motion within one second, then held that position for one second, then transitioned from the end state back to *rest* within one second and held that position for the same amount of time. This process was repeated five times in a sequence for each motion. For each ultrasound image frame in the motion sequence, we computed its distance from the first frame in the sequence. Since the first frame in the sequence corresponds to *rest*, the computed signal represents a deviation from *rest* and movement into a motion. The distance metric was calculated in real-time as *(1-P)*, where *P* is the Pearson’s correlation coefficient between the two frames. A visualization of the signal was provided to participants in real-time as well. Since in the signal areas of low-similarity to *rest* (high muscle deformation) appeared as peaks and areas of high similarity to *rest* (low muscle deformation) appeared as valleys, the participants were able to track the extent to which their muscles were contracted and whether this contraction was consistent over time. Furthermore, using the same signal, we could extract the ultrasound frames corresponding to a motion end state, and the ultrasound frames corresponding to *rest*, by identifying plateaus in the signal as these would occur only where the participant was holding the motion or holding at *rest*. The ultrasound frames at each plateau were averaged into a single representative image and added to a training database with a corresponding motion or *rest* label. The process was repeated until training images corresponding to each movement had been added to the database. The quality of the training database was then determined by performing leave-one-out cross-validation with a 1-nearest-neighbor classifier using Pearson’s correlation coefficient as a similarity measure.

**Figure 2:**
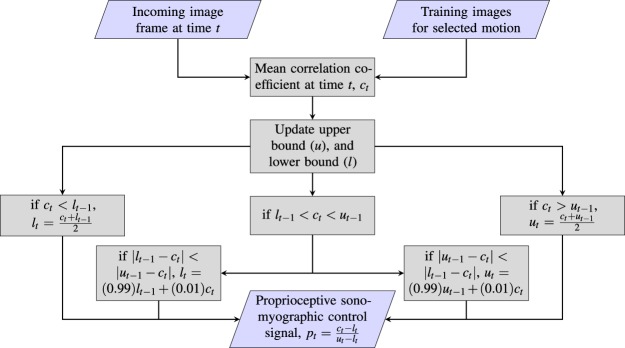
Image analysis pipeline for computing proportional control signal for an incoming, pre-annotated image frame in real-time. Upper bound (*u*) and lower bound (*l*) are initialized as $\infty$ and $-\infty$ respectively.

After training, the participants were asked to perform a specific motion at varying levels of completion while the proportional *proprioceptive sonomyographic control* signal was computed for each incoming frame in real-time. First, all training frames corresponding to the preselected motion were selected from the training database. The mean correlation value, *c*_*t*_, of the incoming image frame at time-point, $t$, versus all frames of the selected motion in the training database was computed. This value was then mapped to a motion completion value between ‘0’ and ‘1’ based on an upper bound, $u$, and a lower-bound, $l$, that were initialized previously during a calibration process. The calibration process was structured in the same way as the training process, only instead of computing the control signal as *(1-P)*, where *P* is the Pearson’s correlation coefficient of the incoming frame to the first frame, the mean correlation value to the trained motion frames, *c*_*t*_, was used instead. The upper bound, $u$, was simply the average signal value at the motion plateaus; while the lower-bound, $l$, was the average signal value at the *rest* plateaus. Since it is highly unlikely that a participant reaches the exact same motion end state and *rest* for every single iteration of a given motion, both bounds were dynamically updated as a weighted sum of the instantaneous correlation signal, *c*_*t*_ and the closest bound. This process is described in detail in Fig. 2, but the general approach to the bound update procedure is as follows: if a correlation signal higher than the expected upper bound was observed, the upper bound was increased; likewise, if a correlation signal lower than the expected lower-bound was observed, the lower bound was decreased; over time, both of the expected bounds were relaxed very slowly, i.e. the expected upper bound was lowered and the expected lower bound was increased, under the assumption that the bounds are uncertain estimates. The effect of the dynamic nature of these updates and the uncertainty of the estimates is a system that is more responsive if we underestimate the initial bounds than it is if we overestimate. An underestimation would result in the participant-controlled end-effector being nudged towards 100% completion, or towards rest, earlier than expected; whereas an overestimation of the bounds would result in the participant being unable to fully reach 0% completion (*rest*) or 100% completion (motion end state). And though we could use fixed bounds and decide not to update the bounds dynamically, we would be risking overestimation and other problems such as variations in the participants muscle activation strength due to fatigue, and variations in signal amplitude due to sensor movements, and environmental noise^34^. Although heuristic, dynamic bound adjustment attempts to mitigate the effect of these factors.

### Experimental protocols

#### Experiment 1 - motion discriminability task

The aim of this experiment was to determine the extent to which our system can distinguish between multiple motions performed by able-bodied subjects and prosthetic users. Participants were asked to perform repetitions of a pre-selected motion, interleaved with *rest* phases between each repetition, in response to audible metronome cues. During the course of the experiment, participants were provided with a view of ultrasound images acquired from their residuum (or intact limb for able-bodied participants) in conjunction with the real-time signal of the current image frame’s deviation from *rest* as described in the previous section (also see video in Supplementary Video M1).

**Figure 3:**
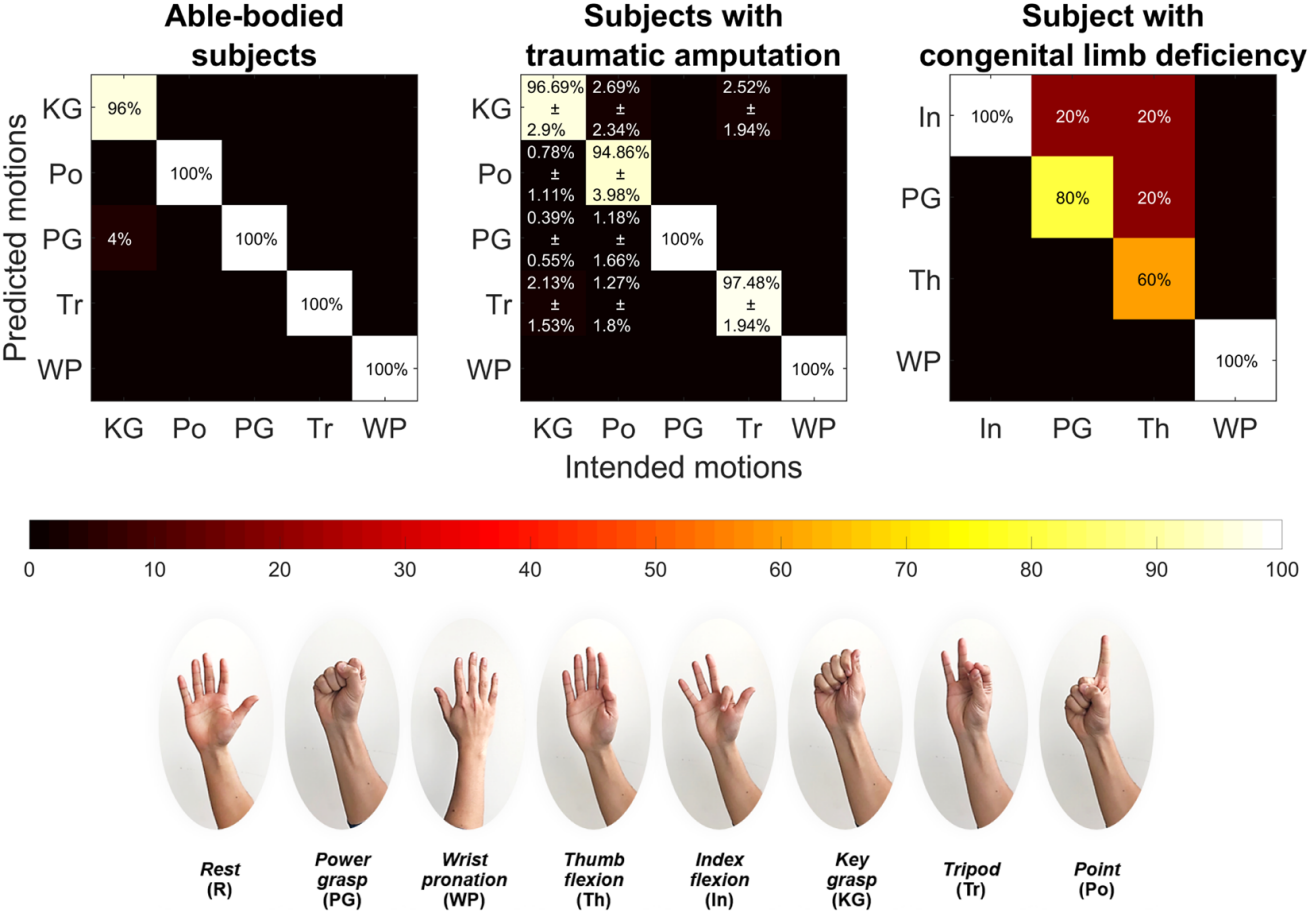
Aggregate confusion matrices showing post-training motion discriminability for able-bodied subjects, traumatic and congenital prosthetic users.

The study involved blocks of trials, each consisting of five repetitions of a predefined set of motions. Trials were repeated until cross-validation (CV) accuracy exceeded 85% and participants reported that they were comfortable performing the motions. All of the participants listed in Table 1 participated in this experiment. Subject-specific motion sets and number of iterations performed by each prosthetic user are listed in Table 3 and motions are pictorially depicted in Fig. 3. All able-bodied participants performed five iterations of *power grasp*, *wrist pronation*, *tripod*, *key grasp*, and *point* each. Outcome measures for this experiment were leave-one out cross-validation accuracies for the first trial block, the best trial block, and the average accuracy over all trial blocks.

**Table 3:** User-intended motions and number of iterations of each motion performed by prosthetic users.

Subject ID	Motions performed	Number of iterations per motion
*Am1*	PG, WP, Tr, KG, Po	20
*Am2*	PG, WP, In, Th	5
*Am3*	PG, WP, Tr, KG, Po	25
*Am4*	PG, WP, Tr, KG, Po	20
Am5*	PG, WP, Tr, KG, Po	15 (S1), 25 (S2)

PG = *power grasp*, WP = *wrist pronation*, Tr = *tripod*, KG = *key grasp*, Po = *point*, In = *index flexion*, Th = *thumb flexion*.

*Motions performed over two different sessions, where S1 = session 1 and S2 = session 2.

#### Experiment 2 - proportional graded control target holding task

The aim of this experiment was to quantify *proprioceptive sonomyographic control* performance of prosthetic users and able-bodied participants at graded muscle activation levels for multiple motions. A motor control task was implemented, where the participant controlled an on-screen cursor that could move up or down in proportion to the degree of muscle activation in the forearm as shown in Fig. 1. The cursor on the computer screen could move up towards a normalized bound of ‘1’ in proportion to the performed completion level of a selected motion, reaching exactly ‘1’ when the motion was at 100% completion. Similarly, the cursor could move down towards the normalized bound of ‘0’ as the user returned from motion completion towards *rest*, reaching ‘0’ when the user was completely at *rest* (see video in Supplementary Video M2).

After an initial calibration step to initialize the bounds, the control interface presented the user with a target position randomly chosen from a predefined set of quantized, equidistant positions (${N}_{P}$), between the normalized upper and lower bounds. The target remained fixed at that position for a set hold-time, ${T}_{H}$, and then moved to the next position until all points were exhausted. For each target position, the participant was prompted to move the cursor to the target by contracting or relaxing their muscles and holding the cursor position until the hold period expired. Participants with unilateral limb loss were also asked to demonstrate the imagined motion, and the extent of flexion using their intact, contralateral arm. Figure 1 shows a screen-shot of the control interface with the target and the user-controlled cursor.

We first conducted a pilot study with *Am2* and *Am3*, with five quantized positions (${N}_{P}=5$) and a hold time, ${T}_{H}=15\,$s to validate our control algorithms. For the pilot study, the hold period commenced when the cursor first entered the current target’s quantization bounds, defined as $Q=5 \%$ of the motion range around the target. If the user failed to enter the quantization bounds within a timeout period, ${T}_{to}=30\,$s, the target automatically moved to the next set-point.

Following the pilot study, all able-bodied participants and prosthetic users *Am3*, *Am4* and *Am5* were recruited to perform the same motion control task; *Am1* and *Am2* were not available. The extended study was performed with eleven graded levels (${N}_{P}=11$). Each target was presented for a fixed amount of time ${T}_{to}={T}_{H}=10\,$s regardless of whether the participant entered the quantization bounds or not. However, the metrics were still calculated after the first time the quantization bounds were entered to avoid including any time that may have been spent transitioning from one target to another. At the end of the 10 s, the target moved to the next target location, randomly chosen from the set of eleven target positions, without replacement. All participants performed three trials of each target level, for a total of 33 target positions.

Position error, stability error, task completion rate, and movement time served as evaluation metrics for *proprioceptive sonomyographic control* performance. Position error was computed as the mean of the difference between the cursor and the target position, while stability error was calculated to be the standard deviation of the difference between the cursor position and the target position. Both, position and stability errors were computed for the time when the cursor first entered the quantization bound $Q$ till the hold time, ${T}_{H}$ expired. Task completion rate was defined as the percentage of targets, out of the total target locations presented, for which the participant was able to successfully reach within $Q$. It is important to note however, that the user was not required to stay within the bound, $Q$, for successful target acquisition. Finally, movement time was calculated as the time from target presentation to when the cursor first entered the quantization bounds $Q$. We performed a Fitt’s law analysis^35–37^ of movement times at various indices of difficulty ($ID$). For experiment 2, $ID$ was defined as in Eq. 1.

1 $$ID=lo{g}_{2}[\frac{{D}_{t}}{2|Q|}+1]$$

where, ${D}_{t}$ is the distance between subsequent targets and $Q$ is the target’s quantization bound.

### Statistical analysis

Due to the extremely small sample size in the study and the difficulty to check the normality assumption required in a MANOVA analysis, we use the one-sided exact Wilcoxon-Mann-Whitney test^38^ to compare the able-bodied group with the prosthetic user group. For the position and stability errors, under the alternative, the distribution in the able-bodied group is shifted to the left of the distribution in the prosthetic user group. On the other hand, under the alternative, the distribution of task completion rate in the able-bodied group is shifted to the right of the distribution in the prosthetic user group.

To compare the effects of the index of difficulty on the movement time between the able-bodied group and the prosthetic user group for the Fitt’s law analysis, we fit the following linear mixed model^39^ including quadratic terms of the index of difficulty ($ID$). For the $i\,$th subject, let ${X}_{ij}$ denote the $j\,$th index of difficulty, ${Y}_{ij}$ the corresponding movement time, and ${G}_{i}=0$ if subject $i$ is in the able-bodied group and ${G}_{i}=1$ otherwise.

2 $${Y}_{ij}=({\beta }_{0}+{\beta }_{1}{G}_{i}+{b}_{i0})+({\beta }_{2}+{\beta }_{3}{G}_{i}+{b}_{i1}){X}_{ij}+({\beta }_{4}+{\beta }_{5}{G}_{i}){X}_{ij}^{2}+{\varepsilon }_{ij}$$

where $\beta \equiv ({\beta }_{1},\mathrm{...},{\beta }_{5})$ denotes the vector of the unknown fixed effects parameters, ${b}_{i0}$ is a random intercept, ${b}_{i1}$ is a random slope, and ${\varepsilon }_{ij}$ is the residual error. Here the two random effects $({b}_{i0},{b}_{i1})$ are assumed to follow a bivariate normal distribution with mean 0 and an unknown variance-covariance matrix. The residual errors ${\varepsilon }_{ij}$ are assumed to be i.i.d. normally distributed with mean 0. Under the null hypothesis that there is no difference between the able-bodied group and the prosthetic user group, we have ${\beta }_{1}={\beta }_{3}={\beta }_{5}=0$.

## Results

### Experiment 1

Our training and prediction strategy was first validated with able-bodied participants. Four out of the five able-bodied participants achieved cross-validation accuracies of 100% within five repetitions (or one trial) across all five motions. Figure 3 shows the aggregate confusion matrix for all able-bodied participants. It shows that four out of five motions were predicted with 100% accuracy and *key grasp* was incorrectly predicted as *power grasp* in just one out of 25 motion instances (5 participants performing 5 motions each).

The same evaluation was then performed with the prosthetic user group. All of the prosthetic users, including the individual with congenital limb deficiency (*Am2*), were able to successfully complete the task, with an average validation accuracy of 96.8 $\pm \,$5.4% for at least 4 motions. Figure 3 shows the aggregate confusion matrix for all subjects with traumatic amputation and the congenital limb deficiency, *Am2*. For subjects with traumatic amputation, the average cross-validation accuracy was 96.76% for five motions, with *key grasp* and *point* having the lowest prediction accuracies. In contrast, *Am2* achieved a cross-validation accuracy of 85% for four motions with *tripod* having the least prediction accuracy at 60%. Having been born without a hand, the participant reported not having clear digit or grasp-based movement context such as the phantom sensation of a thumb movement. This lack of feeling in the residual limb could have negatively affected motion performance and thus also impacted the ability of our system to tell certain motions apart. In fact, the participant had to be guided to attempt to recreate 4 different patterns of movement while looking at the ultrasound for context, instead of being told to imagine his fingers in a specific position like we had done with the other participants. Nonetheless, all participants typically completed the training phase in an hour or less, with relatively high motion discriminability.

We also analyzed the influence of treating *rest* as a separate motion class on the prediction accuracies, instead of treating it simply as the absence of motion (low similarity to motion frames). When *rest* was excluded from cross-validation, motion discriminability improved for all participants. The mean cross-validation accuracy for all trials increased by 7.8% to 96.8% when *rest* was excluded as shown in Table 4. Table 4 also shows cross-validation accuracies with and without *rest* for the initial and best set of trials for prosthetic users. In both cases, the mean cross-validation accuracy for the initial trial was comparable to best accuracy figures showing that the classifier was able to consistently predict motions when presented with representative image frames repeated across several trials.

**Table 4:** Comparison of cross-validation accuracies for prosthetic users with and without *rest* as a motion class.

Subject ID	Cross-validation accuracy (%)					
	Including rest			Excluding rest		
	First trial	Best trial	Average across all trials	First trial	Best trial	Average across all trials
*Am1*	88.33	90.83	87.71	100.00	100.00	99.00
Am2*	80.50	80.50	80.50	85.00	85.00	85.00
*Am3*	82.50	95.00	86.33	96.00	100.00	96.80
*Am4*	100.00	100.00	100.00	100.00	100.00	100.00
*Am5 (S1)*	86.67	86.67	85.00	100.00	100.00	100.00
*Am5 (S2)*	92.50	93.33	89.33	96.00	100.00	96.80
**Mean ± SD**	**89.23 ± 6.82**	**91.49 ± 6.32**	**89.00 ± 6.38**	**96.71 ± 5.50**	**97.86 ± 5.67**	**96.80 ± 5.40**

*Subject performed one trial of five repetitions. Refer to Table 3.

### Experiment 2

**Figure 4:**
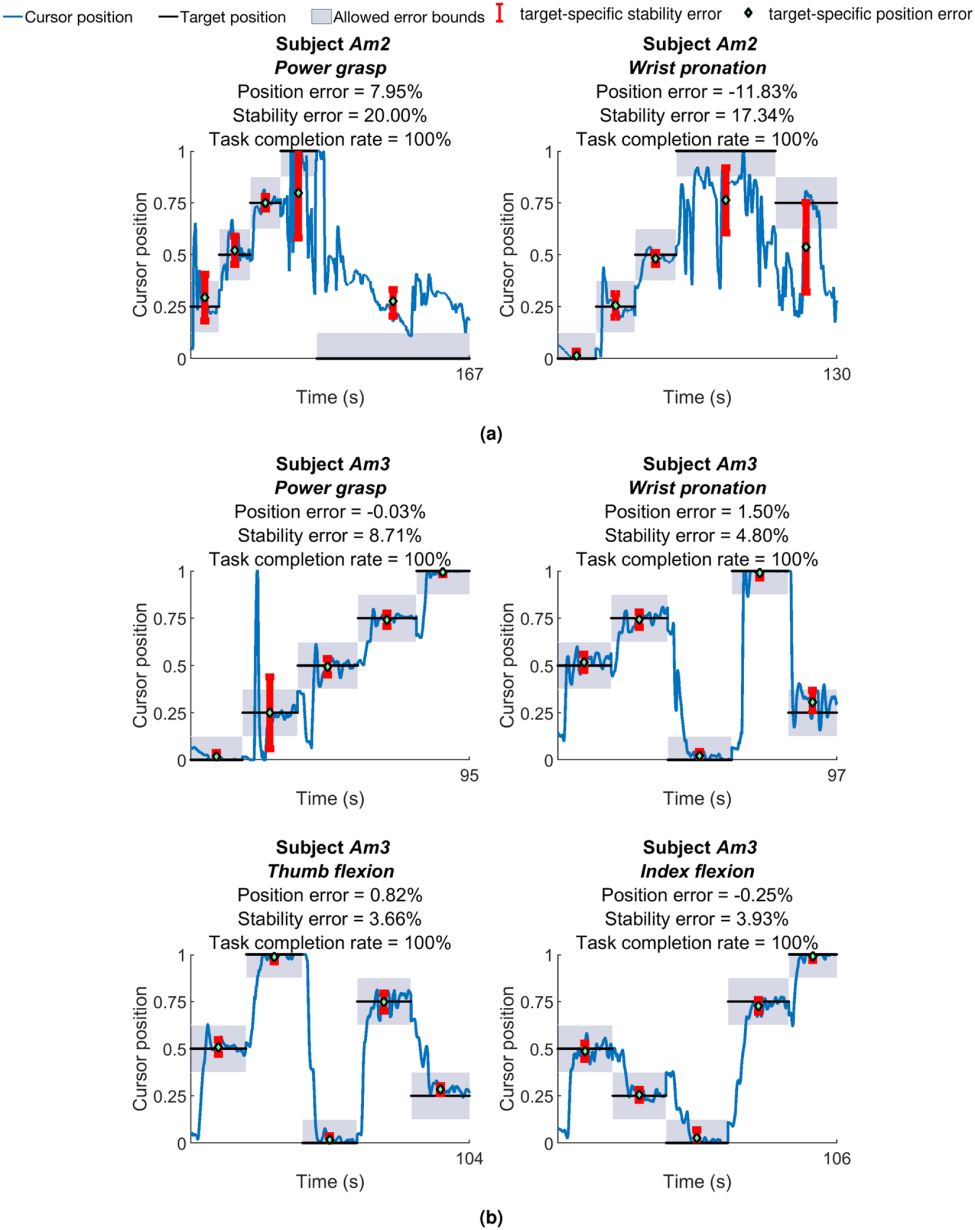
Plots of user-controlled cursor position against target position for the pilot target holding task, for, (**a**) *Am2* and (**b**) *Am3*. The target randomly moved between five quantized target levels within normalized bounds of *rest* (‘0’) and motion completion (‘1’). Position and stability errors for individual target position segments are also shown.

Figure 4a,b show results of the pilot target holding study of experiment 2 for prosthetic users, *Am2* and *Am3,* respectively. The quantized target trajectory, user-controlled cursor position and associated quantization bounds are plotted against time. Both participants were able to successfully reach all of the targets presented in the pilot study. *Am2* performed *power grasp* with position and stability error of 7.95% and 20% respectively. For *wrist pronation* position error was 11.83% whereas stability error was 17.83%. Participant *Am3*’s position errors ranged between −0.03% to 1.50% while stability error ranged from 3.66% to 8.71% across four motions. *Power grasp* had the lowest position error whereas *thumb flexion* was found to have the lowest stability error.

Table 5 shows outcome metrics for the subsequent, extended target holding task performed at eleven graded target levels for able-bodied participants and prosthetic users respectively. All of the able-bodied participants and prosthetic users, *Am4* and *Am5* performed five motions while participant *Am3* performed 2 motions. *Am3* also performed *key grasp*, however, those data were excluded from analysis due to error during the bound calibration stage. The $p$-values of the exact Wilcoxon-Mann-Whitney test are presented in Table 5. Even without multiple comparisons adjustment, no significant difference at the significance level of 0.05 was detected between the two groups for any endpoint of interest with the smallest $p$-value at 0.07. With any procedure for multiple comparisons adjustment, a more stringent significance level is needed; therefore, the conclusion of no significant results remains the same.

**Table 5:** Outcome metrics for target holding task (experiment 2) for able-bodied and prosthetic users.

Subject ID	Position error (%)					Stability error (%)					Task completion rate (%)				
	PG	WP	Po	KG	Tr	PG	WP	Po	KG	Tr	PG	WP	Po	KG	Tr
*Able-bodied Ab1*	−0.74	0.47	0.67	0.63	−0.41	3.66	1.88	4.02	8.45	5.55	93.94	100.0	100.0	87.88	96.97
*Ab2*	1.17	0.41	−0.44	1.21	−0.58	4.90	3.97	1.03	8.87	7.92	90.91	100.0	96.97	96.97	100.0
*Ab3*	−0.66	0.04	0.22	−0.69	−0.38	2.98	1.66	3.52	5.18	9.17	93.94	100.0	81.82	100.0	100.0
*Ab4*	−0.92	−0.25	0.16	1.43	−0.93	3.62	1.62	5.63	9.63	5.97	96.97	100.0	75.76	96.97	81.82
*Ab5*	−1.73	−0.42	0.09	−2.23	−0.57	7.30	4.39	6.78	11.8	7.38	100.0	84.85	96.97	93.94	93.94
**Average**	**1.04**	**0.32**	**0.32**	**1.24**	**0.57**	**4.49**	**2.70**	**4.20**	**8.79**	**7.20**	**95.15**	**96.97**	**90.30**	**95.15**	**94.55**
*Prosthetic user Am3*	2.37	−0.23	—	—	—	10.19	4.61	—	—	—	69.70	100.0	—	—	—
*Am4*	−0.39	−0.25	0.12	−0.49	−0.02	2.33	5.79	3.37	8.55	4.00	96.97	100.0	96.97	96.97	100.0
*Am5*	−6.91	−0.82	−0.01	−5.09	−6.41	13.89	3.92	5.86	15.60	10.90	87.88	100.0	78.79	96.97	100.0
**Average**	**3.22**	**0.43**	**0.07**	**2.79**	**3.22**	**8.80**	**4.77**	**4.62**	**12.08**	**7.45**	**84.85**	**100.0**	**87.88**	**96.97**	**100.0**
**Wilcoxon** ***p***-**value**	0.29	0.52	0.96	0.57	0.57	0.29	0.07	0.57	0.29	0.57	0.91	0.63	0.71	0.52	0.29

PG = *power grasp*, WP = *wrist pronation*, Po = *point*, KG = *key grasp*, Tr = *tripod*.

**Figure 5:**
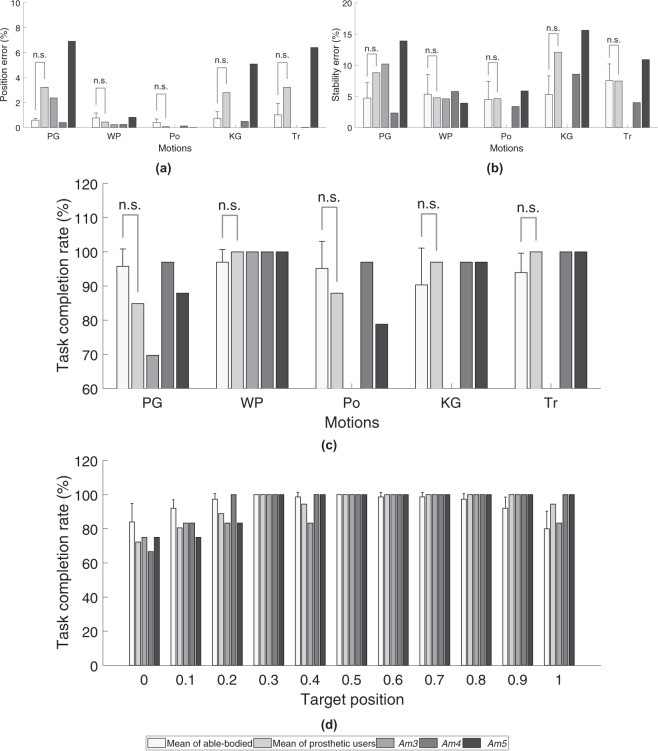
Aggregate outcome metrics for the target holding task (experiment 2) for able-bodied participants and prosthetic users. (**a**) Position error for all motions, (**b**) stability error for all motions, (**c**) task completion rate for all motions and, (**d**) task completion rate at different target positions. Motion legend- PG = *power grasp*, WP = *wrist pronation*, Po = *point*, KG = *key grasp*, Tr = *tripod*.

The average outcome metrics for the target holding task are also shown in Fig. 5. For the targets that were successfully acquired, Fig. 5a,b show that able-bodied participants performed all motions except *point* with lower position and stability errors compared to prosthetic users. Although, mean stability errors for able-bodied participants were lower than prosthetic users, there seems to be a correspondence between motion-specific stability errors across participants. Motions with high stability errors in able-bodied participants also have high stability errors in prosthetic users.

The type of motion also seems to influence position errors and stability errors for all participants, i.e. motions with higher position error also have high stability error and vice versa. For example, as shown in Fig. 5a,b, for able-bodied participants, *key grasp* followed by *power grasp* and *tripod* have the highest stability and position errors. For prosthetic users, a similar trend is observed wherein *key grasp*, *power grasp* and *tripod* exhibited the highest mean stability and mean position errors out of the 5 motions that were performed.

**Figure 6:**
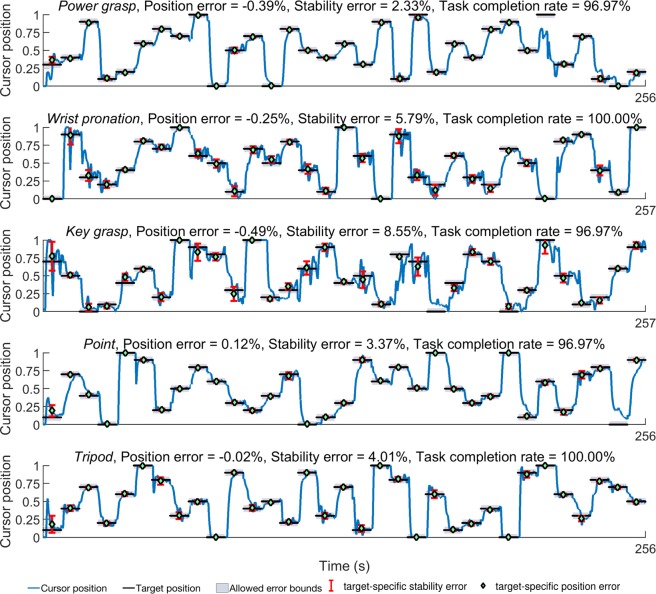
Timeseries plots showing prosthetic user *Am4*’s performance in the target holding task. The user-controlled cursor position and target locations for five motions along with position and stability errors are shown. Motion legend- PG = *power grasp*, WP = *wrist pronation*, Po = *point*, KG = *key grasp*, Tr = *tripod*.

**Figure 7:**
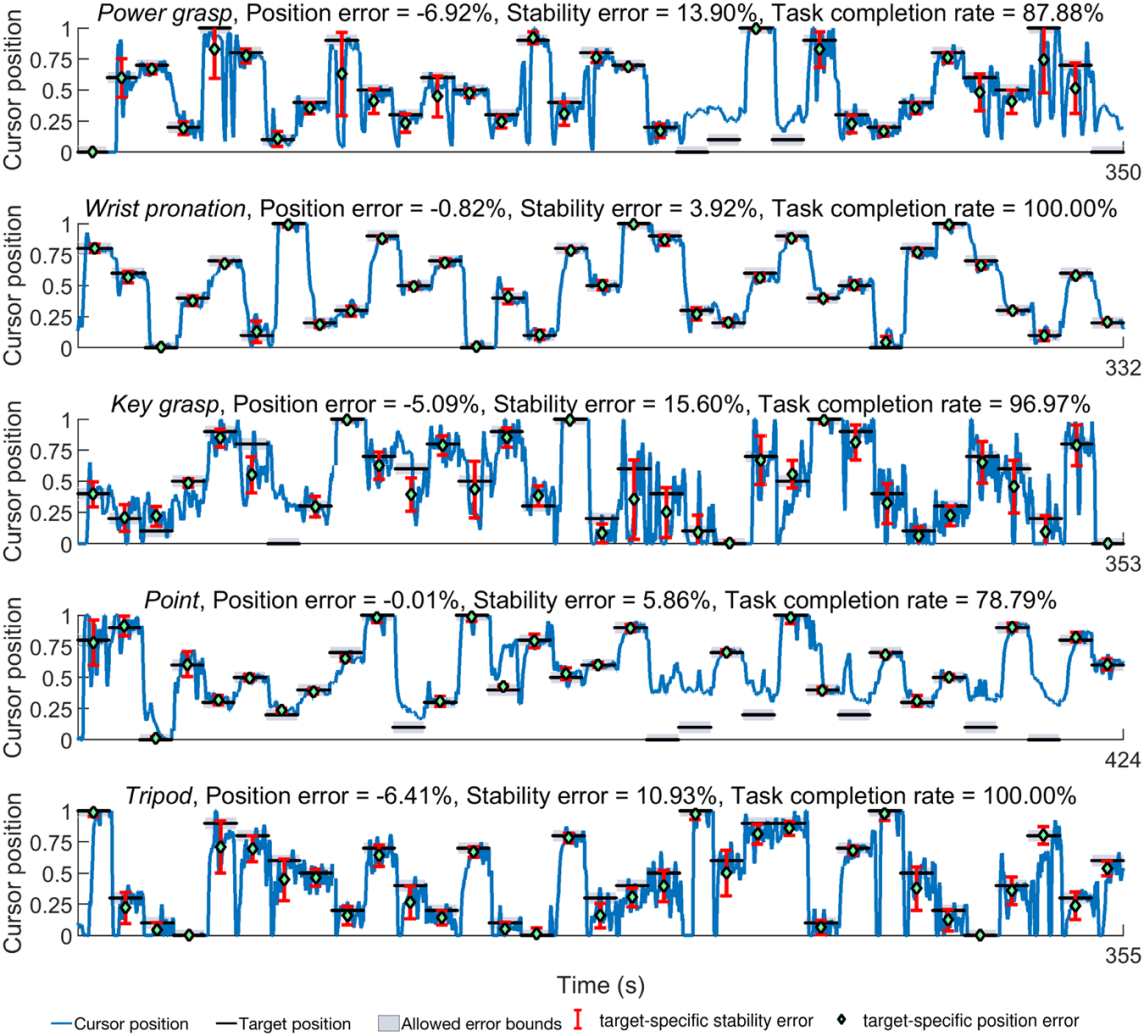
Timeseries plots showing prosthetic user *Am5*’s performance in the target holding task. The user-controlled cursor position and target locations for five motions along with position and stability errors are shown. Motion legend- PG = *power grasp*, WP = *wrist pronation*, Po = *point*, KG = *key grasp*, Tr = *tripod*.

For the extended target holding task, *point* was found to have the lowest average absolute position and stability error followed closely by *wrist pronation* for prosthetic users. However, average task completion rate (Fig. 5c) for *point* was 87.88%, while that of *wrist pronation* was 100% across all trials. Similarly, *power grasp* and *tripod* had comparable position and stability errors, although the average task completion rate was 84.85% for *power grasp* and 100% for *tripod*. Figure 5d also shows that there are small decreases in task completion rates at the lower quartile (0 to 0.25) and upper tenths (0.9 to 1.0) of the total motion completion range for prosthetic users as well as able-bodied participants. Timeseries plots of the cursor and target positions shown in Figs 6 and 7 also indicate that the unachieved targets were mostly presented in the latter half of the task.

**Figure 8:**
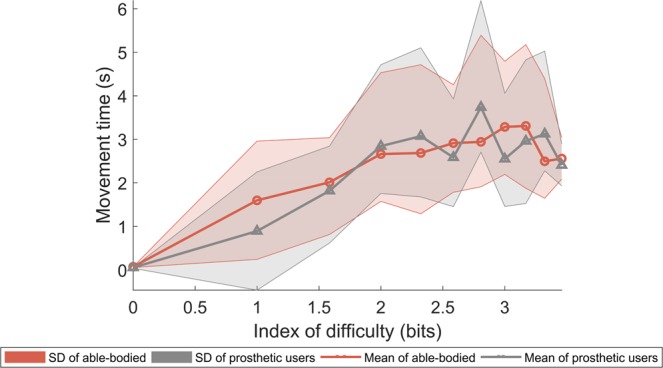
Fitt’s law analysis for the target holding task (experiment 2) for able-bodied participants and prosthetic users.

Finally, results from the Fitt’s law analysis for the movement time required to achieve each target against its corresponding index of difficulty was performed and results are shown in Fig. 8. For both able-bodied participants and prosthetic users, mean movement time increases with increasing task difficulty across all motions as is expected in a human-computer interaction task. A regression analysis was performed of the mean movement times versus index of difficulty for both able-bodied (*R*^2^ = 0.79) and prosthetic users (*R*^2^ = 0.72). Throughputs were found to be slightly higher for able-bodied participants at 1.35 bits/s compared to 1.19 bits/s for prosthetic users. Movement time intercept (y-axis intercept) was also higher for able-bodied participant at 0.72 s compared to 0.44 s for prosthetic users. For the linear-mixed model described in Eq. 2, the likelihood ratio test statistic takes the value of 1.315 leading to a *p*-value of 0.726 compared to the null distribution of ${\chi }^{2}$ with 3 degrees of freedom. Therefore, there is no evidence to show that there is a significant difference between the two groups. The main effects of the index of difficulty and the quadratic term are estimated at ${\hat{\beta }}_{2}=1.654\,[S.\,E.=0.344;95 \% \,CI:(0.980,2.328)]$ and ${\hat{\beta }}_{4}=-\,0.246[S.\,E.=0.083;95 \% CI:(\,-\,0.409,-\,0.083)]$,.

## Discussion

In this paper, we evaluated an ultrasound-based approach for sensing volitional user movement intent and extracting proportional control signals in response to muscle deformations in the forearm. Our proposed *proprioceptive sonomyographic control* strategy allows spatially resolved muscle activity detection in deep seated muscle compartments and extraction of proportional signals from individual functional muscle compartments. Position control based on the extent of muscle deformation is more directly congruent to natural motor control and has the potential to enable robust and intuitive dexterous manipulation that is currently not possible with myoelectric strategies.

### Sonomyography offers position control capability with high number of graded levels

Conventional myoelectric control based on dry, surface electrodes suffer from limited amplitude resolution and hence, achieving multiple graded levels of amplitude continues to be a challenge^10–12^. As a result, myoelectric prostheses employing direct control (DC) utilize simple thresholding of sEMG signals to drive joint velocity as opposed to position. Since the velocity control signal is incongruent with proprioceptive feedback from residual musculature, the ability to intuitively manipulate the terminal device is limited^13^.

In contrast, ultrasound-derived signals have signal to noise >50 dB^29,33^ and can achieve multiple graded levels of amplitude. In previous work, we have shown that ultrasound signals can be proportionally mapped to joint angle^29^. In this study, we evaluated the ability of individuals to achieve true positional control of end-effectors using sonomyography.

Our results show that able bodied individuals and amputees can use sonomyography signals to control the position of a one degree of freedom virtual cursor with 1.10 ± 1.63% position error and 6.11 ± 3.46% stability error on average. All subjects were able to achieve targets multiple graded levels for a number of different motion classes with an average completion rate of 94.98 ± 7.30%.

In this study, we showed that able bodied individuals and amputees can use sonomyography signals to control the position of a 1 degree of freedom virtual cursor, by appropriately flexing their forearm muscles. All subjects were able to achieve multiple graded levels when targets at these levels were presented to them. Due to its one-to-one mapping with ultrasound images of deep seated forearm muscles, the sonomyography signal is a position signal, as opposed to a simple ON/OFF signal like conventional direct control strategies. In the case of velocity control, if an individual wanted the prosthetic hand to acquire a certain partial grasp, they would have to flex their muscles to make the cursor move, then they would have to hold the grasp until the prosthetic hand acquired the required pose, and then rest to make the hand stop moving. In case of position control, the individual would have to just imagine that partial grasp, and the hand would go to that position. This makes position control highly intuitive for users.

### Short training time for high number of separable motions

In this study, users were asked to imagine performing different grasps based on their intuition and any phantom limb sensations. We demonstrated that a simple 1-nearest neighbor classifier with a correlation-based distance metric was able to classify user-intended motions for both able-bodied and individuals with upper-extremity limb loss. This image analysis pipeline makes our system agnostic to anatomical landmarks and removes the need for computationally expensive tracking algorithms^30^. However, due to the nature of our ultrasound-based image analysis pipeline, we must treat rest differently from other motions. The state of muscle deformation at perceived rest positions was found to be quite variable. For able bodied subjects, rest sometimes meant a neutral position with the fingers partially flexed, whereas at other times they extended the digits maximally when asked to rest. For individuals with limb loss, it was even more challenging to standardize a rest position. Fortunately, rest can be classified as a complement to the set of defined motion classes. When rest is eliminated as a potential class for our system to attempt to classify, our approach achieves motion discriminiability that is comparable to current myoelectric pattern recognition (PR) systems performing similar motions^40,41^. Our sonomyography-based approach is able to achieve this classification accuracy with less than an hour of training, compared to several weeks of training needed for PR to achieve comparable classification accuracy^40^.

Nonetheless, it is interesting to note that participants, *Am1*, *Am3* and *Am4* are long-time myoelectric prosthetic users and reported having an advanced level of control over their residual musculature owing to daily use with their prosthesis. This prior experience and repeated practice may have had some influence leading to their initially high cross-validation accuracies as seen in Table 4. This seems to suggest that sonomyography can leverage existing motor skills in current, experienced myoelectric prosthetic users without the need for extensive retraining. Along the same lines, participant *Am5*’s, lower initial accuracy of 86.67%, in the first session (S1) (see Table 4) may have been a result of limited exposure to a powered prosthesis or any other motion performance training. However, in the following session (S2), *Am5* demonstrated a higher initial (92.5%) as well as average accuracy (89.33%) compared to his first session, indicating that the effect of training on our system may have been retained. Additionally, *Am5* later reported an improvement in his ability to control his existing myoelectric prosthesis due to a clearer understanding of his residual muscle deformation in the context of phantom digit movements because of the ultrasound image feedback provided during motion training. Likewise, due to the ultrasound image feedback, the congenital participant *Am2* was able to achieve high motion discriminability for four motions within approximately 30 minutes. This suggests that sonomyography can provide an intuitive control paradigm for individuals with traumatic amputation as well as congenital limb deficiency, who may either lack phantom limb sensations altogether or have limited context of muscular proprioception in their residuum. This is corroborated by contralateral arm demonstrations by subjects with unilateral amputation (see video in Supplementary Video M2) showing that the movements perceived in their phantom limb closely resemble the intended motion.

### Sensorimotor congruency between limb movements and sonomyography

The effect of sensorimotor congruence extends to proportional control task performance as well. We show that participants with traumatic amputation and congenital limb deficiency, with no prior experience of using a sonomyography-based interface are able to demonstrate fine graded control of an end-effector controlled by muscle activity in the forearm. Position errors for prosthetic users and able-bodied participants were below 3.5%, with an average task completion rate higher than 94% for 11 graded targets. Furthermore, despite overall position errors for prosthetic users (1.9%) being higher compared to able-bodied participants (0.7%), there was no appreciable difference in Fitt’s law throughput for both prosthetic users and able-bodied subjects.

We believe that this natural alignment between the behavior of prosthetic users and able-bodied individuals, in the use of our sonomyographic control paradigm is due to the direct mapping between the extent muscle deformation in the forearm and the derived position-based proportional signal and is evidence of the instinctive nature of our strategy. We also demonstrate that the *proprioceptive sonomyographic control* signal can be used to control a commercial prosthetic device (see video in Supplementary Video M3). Subjects *Am*2 and *Am*5 demonstrate that our paradigm provides robust positional control of the prosthetic hand over the entire dynamic range of the motion. Additionally, both subjects clearly show using their contralateral intact limb, that there is a close correspondence between the perceived motion and the motion executed by the prosthetic hand.

Prior studies^42–44^ have shown that individuals using robotic manipulators or prosthetic arms can embody the end-effector into their body schema. Sato *et al*.^42^ demonstrated that congruence between visual and somatosensory inputs can induce a sense of ownership as well as agency in individuals with amputation when using a myoelectric prosthesis. As shown in Supplementary Video M3, with our sonomyographic control paradigm there is a one-to-one mapping from the individual’s muscle flexion and the position of terminal device. While in this study we did not directly attempt to quantify prosthetic embodiment, we believe that *proprioceptive sonomyographic control* may elicit similar effects of embodiment of the terminal device in prosthetic users as observed by Sato *et al*.^42^.

### Limitations of the study

While the results of the current study demonstrate the potential of sonomyography, some limitations should be acknowledged. We did not evaluate simultaneous proportional control of multiple degrees of freedom. While our subjects were able to start using sonomyographic control within a few minutes of training, we did not systematically evaluate the effect of learning and retention over multiple days or weeks. Sonomyography is based on assessment of muscle deformation, which is directly related to the proprioceptive afferents in muscle spindles, however, we did not have any direct measures of proprioception. The subjects received visual feedback of the cursor position and the target position throughout the study. We did not try to separate the effect of visual and proprioceptive feedback. We did not directly compare between sEMG and sonomyography, since position control using sEMG is not practical. We did not include measures of cognitive load in this study. In future studies, we plan to address these limitations.

### Translating sonomyography to practical prototypes

Our current study relied on clinical ultrasound devices for data collection from the participants’ forearms. In order for sonomyography-based approaches to be viable for prosthetic control, the size of the sensing and signal processing systems must be significantly reduced; and although clinical devices continue to be miniaturized and hand-held ultrasound systems are commercially available, the current form factor of commercial systems are still too large to be readily be integrated with commercial prosthetic arms. To overcome this, we are currently developing custom miniaturized and low-power ultrasound imaging instrumentation^45^ that can be directly integrated into a prosthetic shell for continuous, *in-vivo* monitoring of muscle activity and control of associated prosthetic devices. Another concern with sonomyography is the movement of the transducer relative to the anatomy during use or donning and doffing. In previous studies, we have shown that the effect of arm position on classification accuracy can be minimized by adding training samples at different arm positions. Earlier studies with sEMG electrodes integrated into prosthetic sockets have shown that electrode shift, donning/doffing, and arm position can have an adverse effect on long-term control reliability^46–50^. Considering the short training regime required for our approach (typically a few minutes), a re-calibration routine may be a viable solution to retrain the system and overcome sensor shifts. We have also shown in previous studies that the effect of user arm position on classification accuracy for able-bodied participants can be minimized by introducing some variation in training^29^. Although major changes in transducer position will likely severely degrade both classification accuracy and proportional control performance, minor variations in transducer location and orientation due to movement of the residuum inside a prosthetic shell can be mitigated in real-time by appropriate filtering and wavelet-based machine learning techniques^51^. This work establishes the feasibility of an ultrasound-based, noninvasive, muscle activity sensing modality, for use in real-time graded-control of multi-articulated prosthetic arms by individuals with upper-extremity amputation with limited training. Our novel *proprioceptive sonomyographic control* approach provides a means to achieve intuitive proportional control in future prosthetic devices and potentially significantly lower the rate of device rejection.

## Supplementary information

Demonstration of ultrasound probe placement and sonomyographic training routine for prosthetic users.

Virtual cursor control task demonstration of experiment 2.

Demonstration of positional control of commercial prosthetic arm using proprioceptive sonomyographic control by Am2 and Am5.

## Data Availability

The datasets generated during and/or analyzed during the current study are available from the corresponding author on reasonable request.
